# Research on Comprehensive Evaluation and Early Warning of Transmission Lines’ Operation Status Based on Dynamic Cloud Computing

**DOI:** 10.3390/s23031469

**Published:** 2023-01-28

**Authors:** Minzhen Wang, Cheng Li, Xinheng Wang, Zheyong Piao, Yongsheng Yang, Wentao Dai, Qi Zhang

**Affiliations:** 1 National Local Joint Engineering Research Center for Smart Distribution Grid Measurement and Control with Safety Operation Technology, Changchun Institute of Technology, Changchun 130012, China; 2 School of Advanced Technology, Xi’an Jiaotong-Liverpool University (XJTLU), 111 Ren’ai Road, Suzhou 215123, China; 3 Baicheng Power Supply Company, State Grid Jilin Electric Power Co., Ltd., Baicheng 137000, China

**Keywords:** electricity transmission line, exponential scaling method, comprehensive analysis, correlation algorithm, status assessment, cloud computing

## Abstract

The current methods for evaluating the operating condition of electricity transmission lines (ETLs) and providing early warning have several problems, such as the low correlation of data, ignoring the influence of seasonal factors, and strong subjectivity. This paper analyses the sensitive factors that influence dynamic key evaluation indices such as grounding resistance, sag, and wire corrosion, establishes the evaluation criteria of the ETL operation state, and proposes five ETL status levels and seven principles for selecting evaluation indices. Nine grade I evaluation indices and twenty-nine grade II evaluation indices, including passageway and meteorological environments, are determined. The cloud model theory is embedded and used to propose a warning technology for the operation state of ETLs based on inspection defect parameters and the cloud model. Combined with the inspection defect parameters of a line in the Baicheng district of Jilin Province and the critical evaluation index data such as grounding resistance, sag, and wire corrosion, which are used to calculate the timeliness of the data, the solid line is evaluated. The research shows that the dynamic evaluation model is correct and that the ETL status evaluation and early warning method have reasonable practicability.

## 1. Introduction

Maintaining the safe and stable operation of the power system largely depends on the operating status of electricity transmission lines (ETLs) according to relevant industry standards [1,2,3,4,5]. These standards specify the ETL state grades, calculate the score for each grade based on the field-measured data to construct different ETL state quantities, and then obtain the evaluation results. Popoli et al. proposed a reasonable evaluation method that takes into account the influence of non-uniform soil resistivity along the line on the operating state of the line. The results of this method are also relatively accurate, but there is a strong subjectivity [6]. Due to the continuous expansion of the grid scale and improved intelligence, it is not viable to scientifically and comprehensively evaluate the operating conditions of ETLs using only industry standards and the evaluation of single state quantities in the operating state of power systems and ETLs. Adriano M. Junqueira defined several indicators through the AHP, which were combined with a geographic information system (GIS) to determine five types of sensitivity maps, and then to monitor, analyse, and warn of environmental risks to ETLs based on the determined regional and dynamic meteorological and hydrological data [7]. Malhotra proposed a risk-based approach for evaluating and selecting ETL design guidelines in the United States in the event of disruptions caused by hurricanes. The results show that ETLs with longer lines are more economical than with shorter lines [8]. Shafaei et al. proposed a highly effective evaluation method called the Monte Carlo method to evaluate the lightning performance of ETLs [9]. It has been proven that the BP neural network method can assess the damaged faults of ETLs [10], but the model’s computational speed does not meet the requirements of real-time analysis. To address the limitations of these methods, Papia Ray et al. studied the fault type and distance estimation scheme based on a support-vector machine in long ETLs. The forward feature selection method extracts redundant features from the matrix and normalises them. In this way, the variables of the simulation situation dramatically improve the accuracy of ETL fault judgment [11]. While the results are promising, this research is still in its early stages. Additionally, the high-level analysis methods based on the Delphi, questionnaire survey, and expert scoring methods have a significant subjective influence [12,13]. To reduce the personal impact of the evaluation, a method using Bayesian networks has been proposed as a new expert system evaluation approach to evaluate the operation status of ETLs and prove its feasibility [14,15]. There are few studies assessing the operation state of overhead ETLs, so there are still gaps in many aspects [16,17]. Schaefer et al. and Allahvirdizadeh et al. [18,19] introduced an MCDM-based approach to evaluate the performance objectives for the strategic management and development of an energy cloud based on a survey on cloud computing of smart grids. This brings a managerial approach to discussing the objectives, such as resilience, availability, and reliability, in relation to the development of future energy systems, in which ETLs will continue to play a leading role as crucial support points for the new distributed energy systems. Furthermore, according to a previous literature review, the existing research methods are not highly effective, due to the influence of subjective factors. Therefore, it is necessary to study a comprehensive evaluation method that is unaffected by emotional factors [20,21].

The dynamic evaluation model of ETL operation proposed in this paper is intended to evaluate the quality of line state based on inspection defect parameters and data timelines of monitoring status parameters. Through combination with the cloud computing evaluation model, the evaluation results are more accurate and closely align with the actual situation. Moreover, through the addition of the real inspection defect parameters of the Baicheng area, the timeliness of the data status-monitoring parameters and cloud model and the weaknesses, along with the hidden dangers of the transmission lines, can be analysed. Finally, the priority order of line maintenance under the same status level is determined according to the results of the line status level judgment and line status ranking. This model provides supplementary suggestions to operation and maintenance personnel in a timely manner, with reasonable engineering practicality.

## 2. Method for Evaluating ETL Status

This paper integrates a state-level evaluation standard for power systems and combines the improved hierarchical standardisation analysis (HSA) with the CRITIC method by taking into account subjective and objective weights [22]. This enables a more comprehensive, complete, and scientific detection of the ETL status.

### 2.1. Improved HSA Method of Subjective Hierarchical Analysis

**Figure 1:**
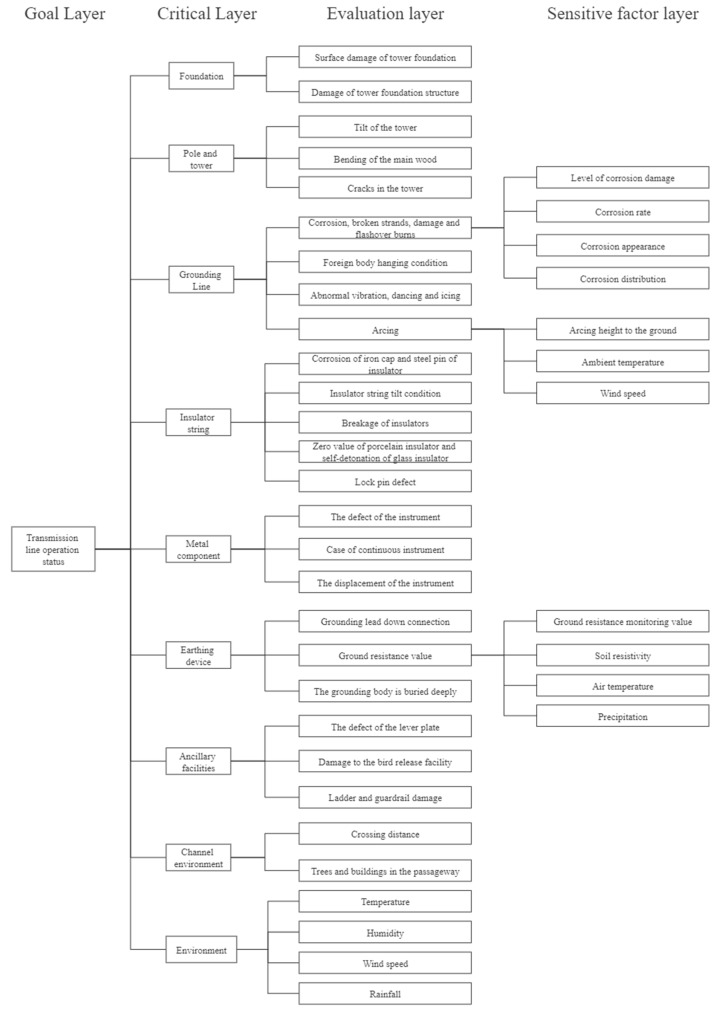
Schematic diagram of the hierarchical model.

Hierarchical analysis was used to subjectively measure the operating status of ETLs corresponding to power standards. In this respect, the active state of the ETL is divided into several state variables, which are further divided into hierarchical state variables and several indicators, ultimately forming a hierarchical structure of the ETL operation index [23,24,25]. A schematic diagram of the hierarchical model is shown in Figure 1.

The key components in the hierarchy are compared and analysed, and the weight coefficient of the ETL’s operating status index is obtained. The specific steps are as follows:

(1) Build a model

Firstly, take the objective-level indicators as the evaluation object. Secondly, decompose the goal-level metrics into a standard-level index based on the division structure of specific standards to form a hierarchical structure model for the subsequent weight distribution.

The overhead ETL status evaluation guide divides the ETL into foundation, tower, grounding wire, insulator string, connector, grounding device, auxiliary facilities, and channel environment. The eight-line element specifies the possible states of each line element. The hierarchical structure model is employed to evaluate the ETL’s operation status, including 8 primary indices and 57 secondary indices.

(2) Construction of the discrimination matrix

Through the cognition and understanding of the hierarchy model, the relationship index between adjacent levels is compared, the membership relationship between each grade is defined, and the discriminant matrix is constructed. The HSA method is improved by the exponential scaling method because it is more in line with the public's thinking, logic, and judgment methods. Exponential scaling also avoids existing problems, such as those found in the three-scale analytic hierarchy process. It addresses issues with dividing the critical relationships between appraisal indicators and reducing the differences in the weight values of appraisal indicators in the 1–9 scale analytic hierarchy process. The exponential scaling method is superior to traditional methods such as the three-scale and nine-scale methods, especially in terms of fitting and scale uniformity [26,27]. The exponential scaling method can be found as shown in Table 1.

Based on the exponential scaling method, the discriminant matrix is constructed as shown in Equation (1):

(1) $$L=\left[\begin{array}{cccc} L_{11} & L_{12} & \cdots & L_{1m}\\ L_{21} & L_{22} & \cdots & L_{2m}\\ \vdots & \vdots & \ddots & \vdots \\ L_{m1} & L_{m2} & \cdots & L_{\mathrm{mm}} \end{array}\right]\;$$

where $L_{\mathrm{ij}}$ is the importance level relative to an index of the i-th index and the j-th index, and the specific standard $L_{ij}=\frac{1}{L_{ji}}$ refers to the importance level described in Table 1.

(3) Consistency check

A consistency check is adopted to minimise the impact of personal factors on the evaluation results, as the AHP is a subjective evaluation method. The consistency check method is as follows: First, the maximum eigenvalue of the discriminant matrix $\lambda_{\max}$ is calculated. Second, the consistency index ${\;C}_{I}$ and ratio index ${\;C}_{R}$ of the discriminant matrix are calculated using Equation (2) and Equation (3), respectively:

(2) $$C_{I}=\frac{\lambda_{\max}-m}{m-1}\;$$

(3) $$C_{R}=\frac{C_{I}}{R_{I}}\;$$

where $\lambda_{\max}$ is the maximum eigenvalue of the discriminant matrix, m is the number of indices at each level, $C_{I}$ is the consistency index of the discrimination matrix, $C_{R}$ is the consistency ratio index of the discrimination matrix, and $R_{I}\;$is the random consistency index. The values of${\;R}_{I}$ are shown in Table 2.

The consistency check is adopted when $C_{R}<0.1$, but if $C_{R}>0.1$, on the other hand, the consistency check is defeated and the discriminant matrix continues the iterative process until the result $C_{R}<0.1$ is obtained.

(4) Index weight calculation

The maximum eigenvalue of the discriminant matrix $\lambda_{\max}$ is calculated from the normalising ω, and the largest eigenvalue of the discriminant matrix and the reassigned indicator weights are obtained. The objective layer indicator and the quasi-lateral layer indicator must construct a discriminant matrix and calculate the indicator weights, respectively. The final subjective weight value α can be acquired through final comprehensive calculations. The calculation formula is as shown in Equation (4):

(4) $$\alpha_{ij}=W_{i}{}^{\left(I\right)}\times W_{ij}{}^{\left(II\right)}\;$$

α: the overall weight of the j-th index in the second level under the i-th index in the first level; $W_{i}{}^{\left(I\right)}$: the weight of the i-th index in level I; $W_{ij}{}^{\left(II\right)}$: the weight of the j-th index in level II under the i-th index in class I.

For the indicator system, the AHP principle can be used to determine the weight by comparing the importance of the indicator layers. According to the membership theory in fuzzy mathematics, the overall early warning level can be determined by synthesising multiple indicator values [28]. However, it cannot consider randomness and ambiguity, and it is difficult to solve complex and fuzzy system problems.

### 2.2. Objective Weight Calculation

The CRITIC method (Criteria Importance Through Intercriteria Correlation) used in this paper considers the extent of variation (i.e., contrast strength) and relevance (i.e., conflict) of indicators, making it a more scientific and comprehensive approach [24,26]. It is important to note that while objective weighting methods often rely on numbers, some of the indicators in Table 1 cannot be directly represented numerically, such as the damage to the tower foundation and the corrosion of the metal foundation. Therefore, it is necessary to convert these indicator states into digital form. This conversion is achieved by dividing the status of the line unit into five levels according to the extent of degradation, including normal, general, attention, abnormal, and authoritarian states, each of which corresponds to a score. The scoring standards are shown in Table 3.

After determining the scoring standard, the CRITIC weighting method can evaluate the objective weight. The steps are as follows:

(1) The standard deviation of each indicator is calculated to reflect the varying extent of each indicator, as shown in Equation (5):
  (5) $$\sigma_{j}=\sqrt{\frac{1}{N}\overset{N}{\underset{i=1}{\sum }}{\left(x_{i}-\mu \right)}^{2}}\;$$
(2) The correlation coefficient $R_{\mathrm{ij}}$ of each indicator is calculated, and the correlation quantification equation $\overset{n}{\underset{i=1}{\sum }}\left(1-R_{\mathrm{ij}}\right)$ is obtained.
(3) The amount of information for each indicator is comprehensively calculated, as shown in Equation (6):
  (6) $$C_{j}=\sigma_{j}*\overset{N}{\underset{i=1}{\sum }}\left(1-R_{\mathrm{ij}}\right)\;$$
  where $C_{j}$ represents the information amount of the j-th index, $\sigma_{j}$ represents the standard deviation, $R_{\mathrm{ij}}$ represents the correlation coefficient, and N represents the number of values of the i-th index.
(4) The index weight β is calculated by Equation (7):
  (7) $$\beta_{j}=\frac{C_{j}}{\sum_{j=1}^{N}C_{j}}\;$$
  where $\beta_{j}$ represents the objective index weight of the j-th index, $C_{j}$ represents the information amount of the j-th index, and N represents the number of values of the j-th index.

### 2.3. Subjective and Objective Evaluation

While both subjective and objective weighting methods have their own advantages, they also have limitations. By combining subjective and objective weights, the disadvantages of a single weighting method can be effectively reduced, and the evaluation results can be made more scientific and comprehensive. The subjective and objective weights are denoted as α and β, respectively. The new combination weight, denoted as γ, can be obtained for the corresponding index. Moreover, the commonly used combination weighting methods are normalised weighting and linear weighting. Normalised weighting, also known as the “multiplier effect”, is mainly applicable in situations with a large number of evaluation indicators and a relatively wide distribution of weights among indicators. It produces results that are smaller when the weights are smaller and larger when the weights are larger. Linear weighting, on the other hand, is a method that weights multiple techniques and has the advantage of producing results with only slight deviation [26]. The calculation formula for the linear weighting method is as follows:

(8) $$W=\sum \eta_{k}W^{\left(k\right)}\;$$

where $\eta_{k}$ represents the weighting coefficient of the k-th index, while $W^{\left(k\right)}$ represents the combined weight value of the k-th index.

Because the linear weighting method is more reasonable, this paper adopts it to calculate the combined weight. Since this research only includes subjective and objective weighting methods, the value of k is 2. The calculation formula is as shown in Equation (9):

(9) $$\gamma_{ij}=\delta \alpha_{ij}+\left(1-\delta \right)\beta_{ij}\;$$

where $\gamma_{ij}$represents the combined weight value of the j-th level II index, $\alpha_{ij}$ represents the subjective weight value of the j-th level II index, $\beta_{ij}$ represents the j-th level II index, and $\gamma_{ij}$ $\alpha_{ij}$, and $\beta_{ij}$ are all under the i-th level I index. For the objective weight value, 0≤δ≤1, considering the high accuracy of the current standard evaluation results, it is only necessary to correct part of the results through objective data. Therefore, δ = 0.7.

### 2.4. Calculation of Evaluation Results

The quantitative data of each evaluation index of the evaluation line are substituted into Equation (10), and then the appraised results of the line’s operating state can be calculated, as shown in Equation (10):

(10) $$U=\overset{i=8,j=n}{\underset{i=1,j=1}{\sum }}\gamma_{ij}\times N_{ij}\;$$

U: ETL operating status score; $\gamma_{ij}$: j-th level II combined weight; $N_{ij}:\;j\text{-}\mathrm{th}$ level II quantitative data; $\gamma_{ij},\;N_{ij}$: the indices under the i-th level I; n: the i-th level index number of level II indicators under level I.

**Figure 2:**
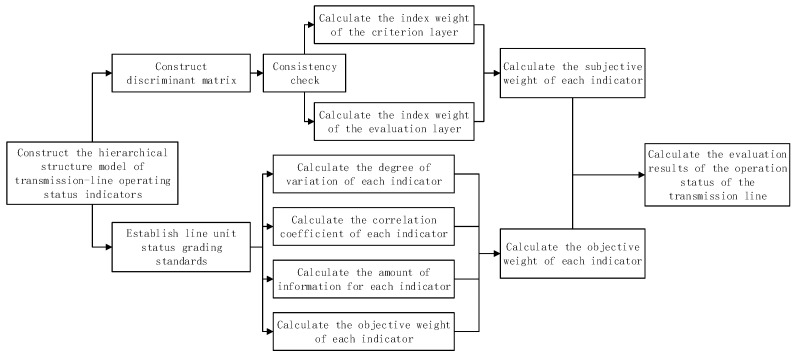
Comprehensive evaluation method of online monitoring.

The process of comprehensive evaluation of the ETL operation status is shown in Figure 2:

## 3. Weight Analysis of Evaluation Indices Based on Expert Experience

### 3.1. Calculation of Subjective Weight

(1) Construct regular discriminant matrix and calculate weight

The literature has shown that an expert questionnaire survey can be used to construct the discrimination matrix of the level I index [23,29,30] using the index scaling method. The classification in Table 1 can be built as shown in Equation (11):

(11) $$A=\left[\begin{array}{cccccccc} 1 & 1/3 & 1.316 & 1/1.732 & 1/1.316 & 1.732 & 2.279 & 3\\ 3 & 1 & 3.947 & 1.732 & 2.279 & 5.194 & 6.836 & 9\\ 1/1.316 & 1/3.947 & 1 & 1/2.279 & 1/1.732 & 1.316 & 3.397 & 2.279\\ 1.732 & 1/1.732 & 2.279 & 1 & 1.316 & 3 & 3.947 & 5.194\\ 1.316 & 1/2.279 & 1.732 & 1/1.316 & 1 & 2.279 & 3 & 3.947\\ 1/1.732 & 1/5.194 & 1/1.316 & 1/3 & 1/2.279 & 1 & 1.316 & 1.732\\ 1/2.279 & 1/6.836 & 1/1.732 & 1/3.947 & 1/3 & 1/1.316 & 1 & 1.316\\ 1/3 & 1/9 & 1/2.279 & 1/5.194 & 1/3.947 & 1/1.732 & 1/1.316 & 1 \end{array}\right]$$

The maximum eigenvalue λ_max_ of matrix A is 8.1568, the largest eigenvector is (0.1078 0.3234 0.0948 0.1867 0.1419 0.0622 0.0473 0.0359)^T^, and the consistency index is calculated according to the maximum eigenvalue $\mathrm{CI}=\frac{8.1568-8}{8-1}=0.0224$. Calculating the consistency ratio indicator based on Table 2, $\mathrm{CR}=\frac{0.0224}{1.41}=0.0159<0.1$. Therefore, the matrix is successfully constructed by passing the consistency check. The weight of the regulation layer can be determined according to the largest eigenvector, as shown in Equation (12):

(12) $$W_{i}^{\left(I\right)}=\frac{w_{i}}{\sum w_{i}}\;$$

$W_{i}^{\left(I\right)}$: weight of the i-th level I index; $W_{i}$: the maximum eigenvector value of the i-th level I index.

The weight of the regulation layer is calculated according to the maximum eigenvector, as shown in Equation (13):

(13) $$W_{\mathrm{ij}}^{\left(\mathrm{II}\right)}=\frac{w_{j}}{\sum w_{j}}\;$$

$W_{\mathrm{ij}}^{\left(\mathrm{II}\right)}$: the weight of the j-th level II index; $W_{i}$: the maximum eigenvector value of the j-th level II index.

Thus, the weights of indicators at the control level are as shown in Table 4:

(2) Appraise layer of the discriminant matrix construct and the weight calculation

The discriminant matrix of each secondary index is constructed according to the standard specifications. This article takes hardware as an illustrative example, as shown in Equation (14):

(14) $$B=\left[\begin{array}{cccccccccccc} 1 & 1 & 1 & 1 & 1 & 1.316 & 3 & 3 & 3 & 3 & 9 & 9\\ 1 & 1 & 1 & 1 & 1 & 1.316 & 3 & 3 & 3 & 3 & 9 & 9\\ 1 & 1 & 1 & 1 & 1 & 1.316 & 3 & 3 & 3 & 3 & 9 & 9\\ 1 & 1 & 1 & 1 & 1 & 1.316 & 3 & 3 & 3 & 3 & 9 & 9\\ 1 & 1 & 1 & 1 & 1 & 1.316 & 3 & 3 & 3 & 3 & 9 & 9\\ \frac{1}{1.316} & \frac{1}{1.316} & \frac{1}{1.316} & \frac{1}{1.316} & \frac{1}{1.316} & 1 & 1.732 & 1.732 & 1.732 & 1.732 & 5.194 & 5.194\\ \frac{1}{3} & \frac{1}{3} & \frac{1}{3} & \frac{1}{3} & \frac{1}{3} & \frac{1}{1.732} & 1 & 1 & 1 & 1 & 3 & 3\\ \frac{1}{3} & \frac{1}{3} & \frac{1}{3} & \frac{1}{3} & \frac{1}{3} & \frac{1}{1.732} & 1 & 1 & 1 & 1 & 3 & 3\\ \frac{1}{3} & \frac{1}{3} & \frac{1}{3} & \frac{1}{3} & \frac{1}{3} & \frac{1}{1.732} & 1 & 1 & 1 & 1 & 3 & 3\\ \frac{1}{3} & \frac{1}{3} & \frac{1}{3} & \frac{1}{3} & \frac{1}{3} & \frac{1}{1.732} & 1 & 1 & 1 & 1 & 3 & 3\\ \frac{1}{9} & \frac{1}{9} & \frac{1}{9} & \frac{1}{9} & \frac{1}{9} & \frac{1}{1.5194} & \frac{1}{3} & \frac{1}{3} & \frac{1}{3} & \frac{1}{3} & 1 & 1\\ \frac{1}{9} & \frac{1}{9} & \frac{1}{9} & \frac{1}{9} & \frac{1}{9} & \frac{1}{1.5194} & \frac{1}{3} & \frac{1}{3} & \frac{1}{3} & \frac{1}{3} & 1 & 1 \end{array}\right]$$

The maximum eigenvalue λ_max_ of matrix B is 12.0518, and the largest eigenvector is (0.1378 0.1378 0.1378 0.1378 0.1378 0.0918 0.047 0.047 0.047 0.047 0.0157 0.0157)T. The consistency index can be calculated based on the largest eigenvalue, as follows: $\mathrm{CI}=\frac{12.0158-12}{12-1}=0.0014$. According to Table 2, the consistency ratio index can be calculated as follows: $\mathrm{CR}=\frac{0.0014}{1.54}=0.0009<0.1$ This indicates that the discriminant matrix has been successfully constructed and that the consistency check has been passed.

The weights of each index for the line unit pipe fitting evaluation layer are shown in Table 5.

(3) Overall weight calculation of each indicator of the appraise layer

The weight of each index that determines the value of the appraise layer can be calculated using Equation (4), using the value of the fitting as an example. The index of each line element that contributes to the appraise layer is presented in Table 6.

Each of the line unit weight global evaluation index layers (subjective weight) based on the calculation process is shown in Figure 2.

### 3.2. Objective Weight Calculation

The standard deviation indicates the degree of variation in the dispersion area of each index. By substituting the scores of the operating status of the six ETLs into the standard deviation formula, the degree of variation for each index can be determined. The Kendall correlation coefficient is then used to calculate the correlation between the indicators. The Kendall correlation coefficient is a reliable measure that is often used to assess the level of consistency in scoring data. It is often used to study the consistency levels of scoring data and then substitute each indicator’s corresponding degree of variation and correlation coefficient into Equation (6) to obtain the amount of information. The objective weight of the index can be obtained through the combination of these calculations, as shown in Equation (15):

(15) $$M=\frac{S}{\frac{1}{12}\left[K^{2}\left(N^{3}-N\right)-K\sum_{i=1}^{K}T_{i}\right]}\;$$

where N is the number of objects being evaluated. There are K total evaluators or scoring criteria, and S is the sum of the square deviations of the scores for each subject, with the average of all of these sums being calculated.

According to the calculated results, the maximum positive correlation coefficient is 0.943, while the highest negative correlation coefficient is −0.447. Therefore, the correlation coefficient range of each indicator is [−1, 1]. Figure 2 illustrates the degree of variation and correlation of each indicator, including both quantitative and computational results.

Examining the Table 7 data, it can be seen that there are numerous 0s in the final results because, in Equation (5), the average value and standard deviation of each indicator are calculated. If the state of a specific indicator for the ETL remains unchanged, the index will not change. However, if the indicator state is scored multiple times, the standard deviation will be 0 when calculated using Equation (5). For example, in this round of investigation, the intersection distance of the six lines is 5 points, resulting in an average score of 5 for this indicator. Therefore, the standard deviation calculated using Equation (5) is 0. Many objective data evaluation methods [31,32] form the foundation of the CRITIC method. A larger volume of data can make the evaluation results more scientific and comprehensive, while a small or insufficient amount of data may result in a weight value of 0.

### 3.3. Calculation of the Weight

**Figure 3:**
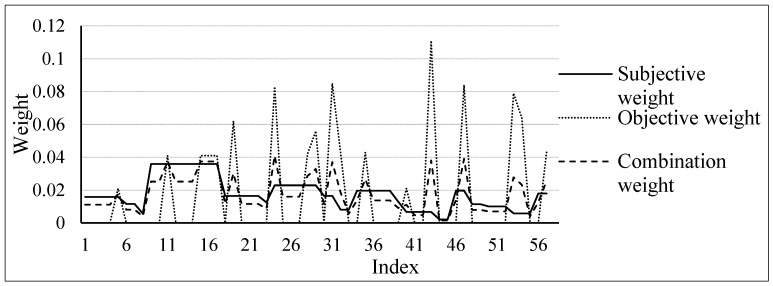
Comparison result chart of subjective weight, objective weight, and combined weight.

The complete weight calculation results for each index and the comparison of different weights can be seen in Figure 3. Using the subjective weight and objective weight values for each index and Equation (9), the comprehensive weight value for each index is calculated:

Judging from the outcome of the combined weights, the method of combining subjective and objective weights can effectively prevent imbalances in the objective weights. Under different subjective and objective index weights, the weighting value of 0 does not occur. For example, when the supervisor weight and objective index weighting are 0.0158 and 0, respectively, the weight obtained by the combined weighting method is 0.0111. On the other hand, when there is a significant discrepancy between the results of subjective and objective indicators, relying solely on personal evaluation can be highly subjective and may not accurately reflect the actual situation. For example, when the emotional weight of the index is 0.0067, the impact is minimal, but the objective weighting result is 0.111. This has led some scholars to conclude that the effect of this index is minimal, even though it may appear more frequently in practice.

## 4. Evaluation of Cloud Model Establishment and Verification

Early warning involves issuing notifications and alarms in advance based on the signs of potential risks before the risk occurs, in order to mitigate potential dangers. Multi-indicator comprehensive early warning involves analysing multiple indicators that pose a threat to the security of the system through a mathematical model, comparing the analysis results to an alarm threshold, and issuing an early warning based on the comparison results.

### 4.1. Applicability of the Cloud Model in Early Warning of ETL Operation Status

Multi-indicator comprehensive early warning is useful for gaining a comprehensive understanding of the overall risk level of the evaluation target and taking timely preventive measures, which can significantly reduce losses. The principles and characteristics of standard multi-index comprehensive early warning methods are shown in Table 8.

The above three methods have their own advantages. The cloud early warning model can effectively evaluate both qualitative and quantitative indicators and generate early warning levels by taking into account randomness and fuzziness. In comparison to fuzzy comprehensive evaluation, this method can reduce the subjectivity in the evaluation process more effectively and improve the accuracy. Therefore, the cloud model is the most applicable for early warning of the ETL’s operation state among the previously mentioned methods.

The early warning levels of the ETL’s operating status are divided into five groups: normal, attention, alarm, abnormal, and severe. Furthermore, the generated ETL operating status standards are entered into the evaluation cloud model, and only the operation status index evaluation cloud and comprehensive evaluation cloud of the ETL [41,42,43,44] need to be determined, after which the state early warning analysis can be conducted.

### 4.2. Evaluation of the Cloud Model and the Platform’s Establishment

Compared to the fuzzy set theory that is currently commonly used, the application of cloud model theory in evaluating ETL status can utilise the advantages of randomness while accounting for ambiguity [45,46]. In this research, qualitative and quantitative indicators can be transformed into one another. The cloud model proposed in this study is an image composed of numerous “cloud drops”. When the level of blurriness is high, the merged image appears similar to a “cloud”, and when the level of blurriness is low, the combined image appears like a “curve”.

(1) Theory of the cloud

In evaluating the cloud model, firstly, let X be a quantitative dataset, i.e., $X=\left\{x\right\}$. This quantitative dataset is called the universe of discourse. C is a qualitative concept on the universe of discourse X, and if x∈X is a random map of $\mu \left(x\right)\in \left[0,\;1\right]$, then the distribution of x on X is called a cloud, denoted as $C\left(x\right)$. $\mu \left(x\right)$ is the cloud x of membership to C, and each (x, $\mu \left(x\right)$) is called a cloud drop, denoted as a drop (x, $\mu \left(x\right)$). The corresponding mathematical relationship is as follows:

(16) $$\mu :X\to \left[0,1\right],\;\forall x\in X,\;x\to \mu \left(x\right)\;$$

Through the establishment of the cloud model, it can be seen that the cloud model has the following characteristics:

Firstly, cloud droplets are a random realisation of the transformation of qualitative concepts into quantitative data, so the process of generating cloud droplets is the process of mutual adaptation between quantitative and qualitative concepts. This process is also a manifestation of randomness and fuzziness [47,48]. Secondly, the more cloud droplets there are, the clearer the generated cloud becomes, and the more accurately the qualitative concept can be expressed.

(2) Digital Characteristics of Clouds

The cloud model mainly uses three numerical features: expected value Ex, entropy En, and super entropy He, denoted as C (Ex, En, He). These numerical features are also an intuitive manifestation of qualitative concepts. The determination of each digital component is as follows:

- Expectation (Ex): This refers to the expectation of cloud droplet distribution in the universe of discourse, and it is also the core of a cloud, meaning the most probable point of a qualitative concept in the universe of discourse.
- Entropy (En): This measures the randomness of qualitative concepts, which reflects the extent of dispersion of a cloud drop. Furthermore, it reflects the acceptable range of cloud drop values in the universe of discourse. Overall, the value of En directly determines the width of a cloud.
- Super entropy (He): This reflects the uncertainty of entropy, or the entropy of entropy, and its value determines the thickness of a cloud. A high value of He corresponds to high dispersion and viscosity of the cloud.

**Figure 4:**
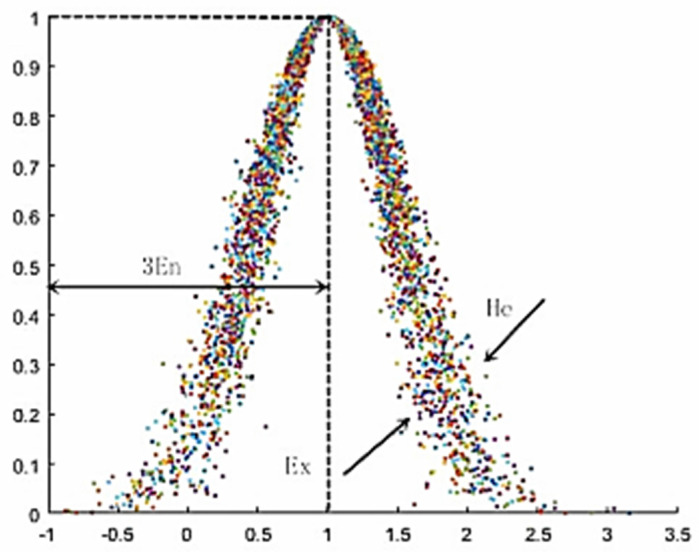
Cloud model digital feature scheme.

In this case, when Ex = 1, En = 0.5, He = 0.08, and the number of cloud droplets (n) = 1500, the digital feature diagram of the generated cloud model is as shown in Figure 4.

(3) Clouds computing model platform establishment

The platform collects cloud droplets through the cloud generator—a tool that realises the mutual conversion of qualitative and quantitative data, divided into forward and reverse cloud generators.

The forward cloud generator is vital for converting the digital features C (Ex, En, He) into cloud droplets (x,) and further generating cloud images.

Input: Numerical features C (Ex, En, He) and the number of cloud droplets n.

Output: n cloud droplets xi and their quantitative values.

The steps of the forward cloud generator algorithm are as follows:

- Generate a standard random number En’ with En as the expectation and He as the standard deviation;
- Generate a regular random number xi with Ex as the expectation and En’ as the standard deviation;
- Calculate the cloud titre value using Equation (17):
  (17) $$\mu \left(x_{i}\right)=e^{-{\left(x_{i}-Ex\right)}^{2}/2{\left(En\right)}^{2}}$$
  Then, (xi,$\mu \left(x\right)$) is a cloud droplet, which realises the conversion of qualitative concepts into quantitative concepts;
- Repeat steps a–c n times to generate a sufficient number of cloud droplets.

The reverse cloud generator is the inverse operation of the forward cloud generator. By inputting a known number of cloud droplets that have been generated by drop (x,$\mu \left(x\right)$), the output is a digital feature C (Ex, En, He).

Input: n cloud drops xi.

Output: Numeric features C (Ex, En, He) representing n cloud centricity concepts.

The steps of the reverse cloud generator algorithm are as follows:

- Input n cloud droplets xi, and calculate the mean value of this group of cloud droplets—that is, the cloud model digital feature expectation Ex and the sample variance S2—using Equations (18) and (19):
  (18) $$\bar{X}=\frac{1}{n}\sum_{i=1}^{n}x_{i}$$
  (19) $$S^{2}=\frac{1}{n-1}\sum_{i=1}^{n}{\left(x_{i}-\bar{X}\right)}^{2}$$
- Calculate the digital feature entropy En of the cloud model using Equation (20).
  (20) $$En=\sqrt{\frac{\pi }{2}}\cdot \frac{1}{n}\cdot \sum_{i=1}^{n}\left|x_{i}-\bar{X}\right|$$
- Calculate the digital feature super entropy (He) of the cloud model using Equation (21).
  (21) $$He={\left(S^{2}-En^{2}\right)}^{\frac{1}{2}}\;$$

In this research, the platform classifies the early warning level of ETL operation status into five levels: “normal state”, “attention state”, “warning state”, “abnormal state”, and “severe state”. The scoring interval is set at [0, 10]. For example, “severe state” corresponds to the interval [0, c1], “abnormal state” corresponds to the interval [c1, c2], “warning state” corresponds to the interval [c2, c3], “attention state” corresponds to the interval [c3, c4], and “normal state” corresponds to the interval [c4, 10]. The distribution of score intervals is shown in Table 9.

The digital eigenvalues C (Ex, En, He) of the cloud model for each early warning level are calculated using the bilateral constraint method. The calculation process is as shown in Equation (22):

(22) $$\left\{\begin{array}{l} Ex=\left(C_{\min}+C_{\max}\right)/2\\ En=\left(C_{\max}-C_{\min}\right)/6\\ He=k \end{array}\right.\;$$

where C_min_ represents the lower bound of the scoring interval, C_max_ represents the upper bound of the scoring gap, and k is a constant that determines the thickness of the cloud.

The standard evaluation cloud C (Ex, En, He) can be generated by inputting the cloud model digital eigenvalues C (Ex, En, He) of each evaluation level and the set number n of cloud droplets into the forward cloud generator.

Based on expert experience and relevant standards and specifications for ETL status assessment, this paper determines the early warning level scoring interval, and the distribution of the scoring gap is as follows: “Severe state” corresponds to the interval [0, 2], “abnormal state” corresponds to the interval [2, 4], “warning state” corresponds to the interval [4, 6], “attention state” corresponds to the interval [6, 8], and “normal “state” corresponds to the interval [8, 10].

The calculation results for the digital eigenvalues of the cloud model for each evaluation level, as determined by Equation (22), are shown in Table 10.

### 4.3. Evaluate the Impact of Cloud Model Dynamic Weight

(1) Determine the index and evaluate the cloud

**Figure 5:**
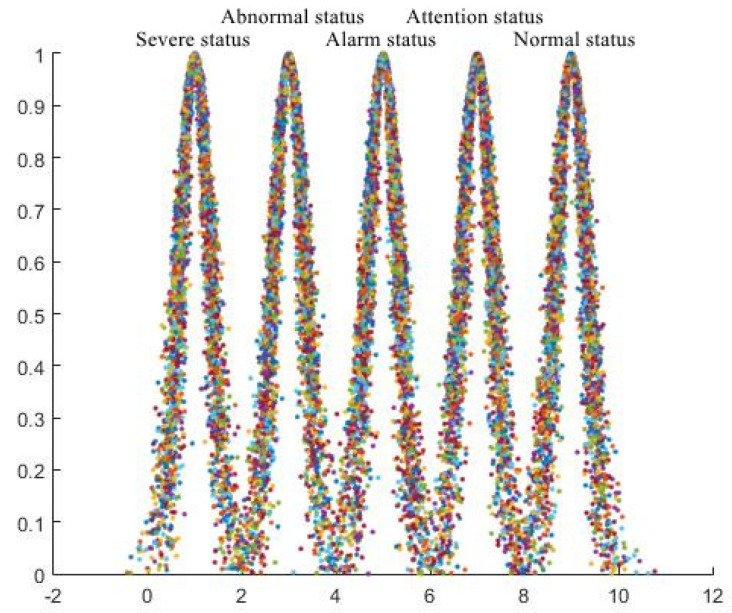
Regulation layer dynamic index evaluation cloud (the digital eigenvalues of cloud models, including each evaluation level, are calculated by the forward cloud generator, and the number of raindrops is set to n = 5000).

Given the status rating standard of the ETL operation status evaluation index described in Table 1, the ETL operation status evaluation index can be summarised as a qualitative index. Therefore, through combination with the method for determining a qualitative index evaluation cloud from a previous article, a regulation stratus cloud model with dynamic characteristics can be generated [49,50]. Furthermore, through inputting the digital eigenvalues of the cloud model for each evaluation level into the forward cloud generator, and setting the number of raindrops (n) = 5000 according to the early warning level classification of the cloud computing platform, a generated regulation layer dynamic index evaluation cloud can be constructed, as shown in Figure 5.

(2) Determining the criteria layer of the dynamic index evaluation cloud

**Figure 6:**
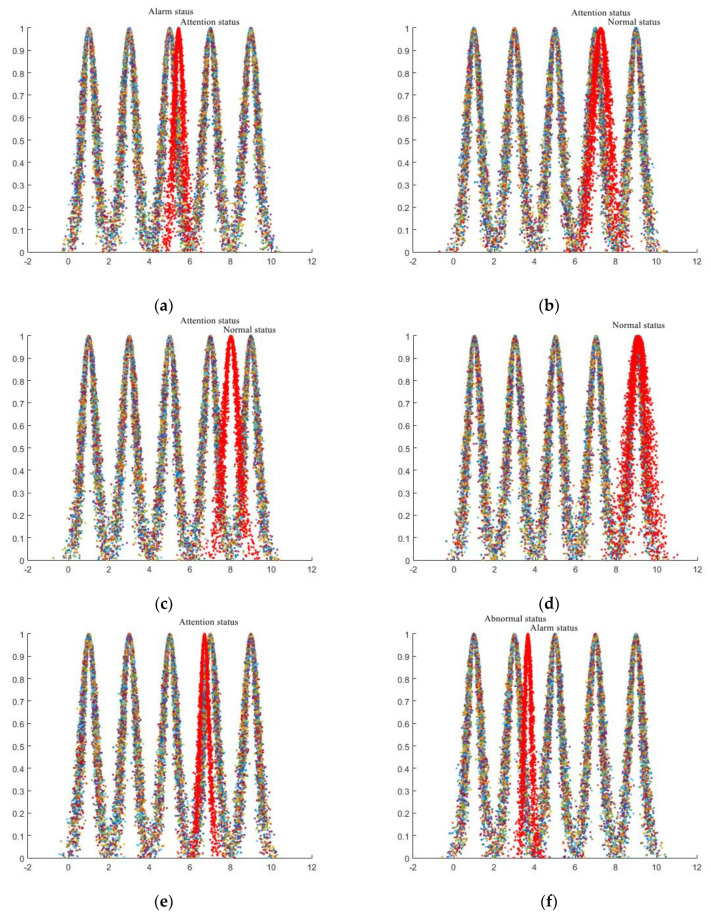

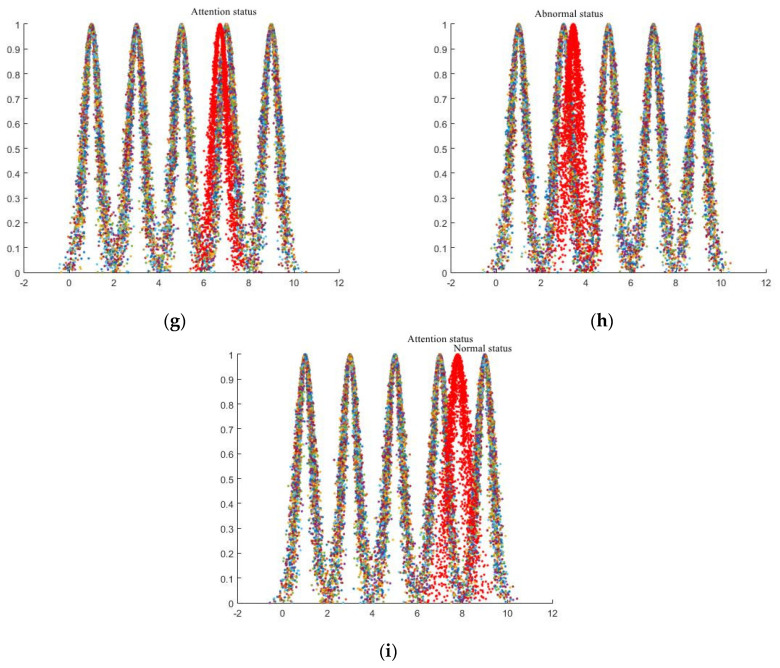
Multiple main objectives affecting the line status are evaluated through the cloud computing platform’s standard-layer dynamic indicators. (**a**) Grounding fittings. (**b**) Auxiliary facilities. (**c**) Towers. (**d**) Foundation. (**e**) Grounding devices. (**f**) Auxiliary facilities. (**g**) Insulator strings. (**h**) Meteorological environment. (**i**) Channel environment.

By inputting the characteristics of indicators (i.e., foundation, towers, ground conductors, insulator strings, grounding fittings, grounding devices, auxiliary facilities, channel environment, meteorological environment) that mainly affect the line status of each regulation layer into the digital cloud generator of the forward cloud model and setting the number of clouds drops to n = 2300, the evaluation cloud of each regulation layer index can be obtained. The specific situation is as shown in Figure 6.

(3) Determine the dynamic, comprehensive evaluation cloud

**Figure 7:**
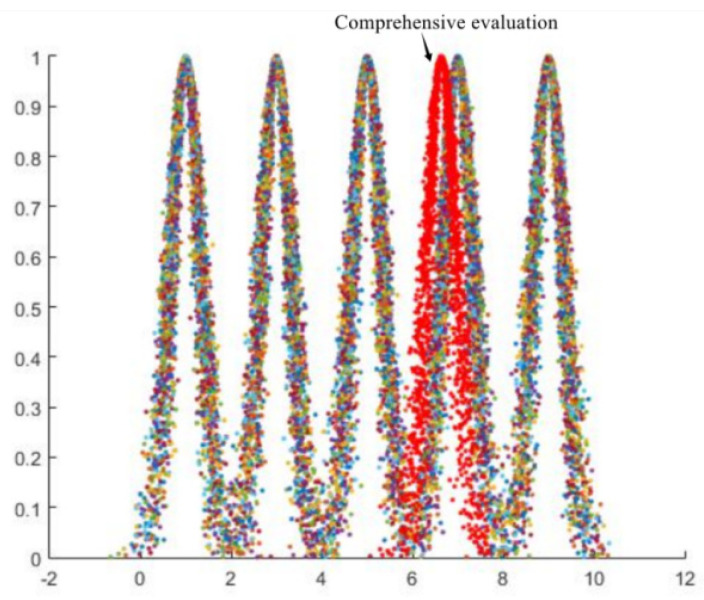
The final evaluation cloud map of the comprehensive multi-index evaluation.

The weight value of each regulation level can be calculated according to the evaluation index combination weight listed in the evaluation standard. This is achieved by summing the corresponding evaluation layer index combination weight under each regulation level to obtain the regulation level weight value. Based on the calculation results influenced by various factors shown in the figure above, a comprehensive digital evaluation cloud featuring the ETL operating status is generated. By inputting the digital element into the forward cloud generator and setting the number of clouds to drop n = 2300, the generated comprehensive evaluation cloud is as shown in Figure 7.

The primary state is “normal”, the towering state is “normal”, the ground wire state is “alarm”, the insulator state is “attention”, the appropriate state is “abnormal”, the grounding device state is “attention”, the status of the ancillary facilities is “attention”, the channel environment status is “attention”, and the meteorological environment status is “abnormal”. It can be realised that the tower’s foundation is in good condition, but the insulators, grounding devices, auxiliary facilities, and the passage environment have certain hidden dangers that do not affect the regular operation of the line monitoring of indicators at each appraised level of the ground wire. Nevertheless, the state of the fittings endangers the stable operation of the line and requires prompt maintenance to ensure the regular operation of the line. The meteorological environment also influences the line state, but it is an external factor that cannot be controlled by humans. Therefore, it is essential to prevent the impact of meteorological climate in advance, and to strengthen the monitoring and timely maintenance of other line indicators.

## 5. Results and Discussion

According to the actual situation, the has–CRITIC combined weight method has been improved (as shown in Equation (10)). The operating status scores of the six ETLs can be obtained by substituting the data.

### 5.1. Evaluation Indices’ Dynamic Weight Determination Based on Expert Experience

The cloud computing evaluation data are compared with the HSA method, the improved has–CRITIC method, and the standard specification. In this paper, the CRITIC method is compared to the traditional deductive systematic evaluation method, and the comparison results are shown in Table 11.

The results shown in Table 11 indicate that when evaluated according to the standards, the six ETLs’ health status is ranked as #1 > #3 > #5 > #4 > #2 > #6, but when using the improved HSA–CRITIC method proposed in this paper, the health state of those lines is ranked as #1 > #3 > #2 > #5 > #4 > #6. It can be seen that the best and worst results of the two evaluation methods are consistent, indicating that the enhanced HSA–CRITIC process has a certain practicability in the short term. Nonetheless, the standard specification can only display the evaluation status; the specific advantages and disadvantages of lines #1, #3, #4, and #5 cannot be broken down. When encountering a situation where the pros and cons of these four lines need to be evaluated, the evaluation methods of the standard specifications cannot be applied, demonstrating the limitations of relying on traditional standards and specifications to assess line status. Furthermore, the six lines determined according to the HSA method and the improved HSA method are ranked in the same order of pros and cons, which is #1 > #3 > #5 > #2 > #6 > #4, but the specific scores are different. The index scaling method of the improved HSA method is more in line with psychological conditions.

Therefore, when the conditions are met, the improved HSA method is more reasonable than the traditional HSA method. However, whether it is the conventional HSA method or the improved HSA method, their advantages and disadvantages differ from the standard and standardised evaluation results. The evaluation results of the CRITIC method are also different. For example, in the case of line #6, according to the expert evaluation method, it is rare for insulators to have severe problems simultaneously. However, according to the improved level evaluation and correlation analysis, there are three-line insulator strings, which will be judged as severe fault problems, significantly impacting the line. This demonstrates that it is impossible to scientifically and comprehensively evaluate the line status solely by relying on the subjective judgment of experts. The CRITIC method ranks the lines as #1 > #3 > #4 > #2 > #5 > #6, which is similar to the standard and standardised evaluation results, but it is unreasonable to evaluate based on objective data alone. For example, the indicator $T_{62}$ line #2 has three consecutive bases, or more than the specified value, which causes the objective weight to be too large. In the opinion of experts, due to daily maintenance of the grounding device, there should be minimal serious problems, so the emotional weight of experts is higher. Based on the above analysis, the improved HSA–CRITIC method subjectively scores the line from a perspective that is more in line with the psychological state and, at the same time, modifies the subjective score based on objective data. This method avoids the unbalanced weight when assigning a single accurate weight, while retaining experts’ personal opinions. Therefore, the improved HSA–CRITIC method enables a more scientific and comprehensive evaluation of the ETL’s operation condition compared to the previous process.

### 5.2. Analysis of Sensitive Influencing Factors of Some Key Evaluation Indices, including Data Timeliness

Since most transmission lines are set up in the outskirts of the country where there are few people, the operating state of the lines is greatly affected by natural environmental factors, so it is necessary to analyse the sensitive influencing factors of the theoretical algorithm. Combined with the characteristics of the complex climatic environment in Northeast China, we selected soil resistivity, earth resistance, micrometeorology, and traverse sag state affected by seasons to revise the theoretical model.

**Figure 8:**
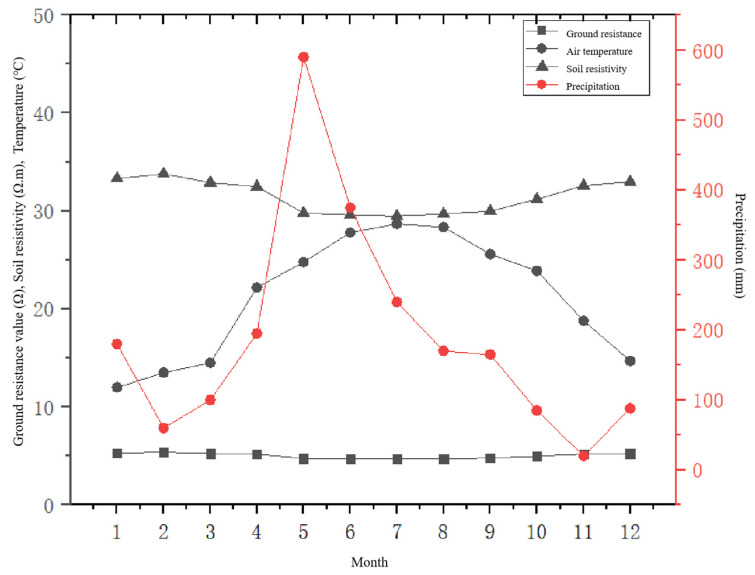
Monthly variation relationships between earth resistance and soil resistivity, temperature, and precipitation.

The results of the study showed in Figure 8 that there were noticeable increases in precipitation and temperature from February to June, resulting in decreases in soil resistivity and ground resistance, and these values reached their minima in July and August. Precipitation and air temperature began to decrease gradually from June to November, while soil resistivity and ground resistance gradually increased, reaching their maximum values in December and January. It appears that the ground resistance exhibits a clear trend of change with seasonal changes, and it is positively correlated with soil resistivity changes and negatively correlated with temperature and precipitation changes. The monthly minimum values of earth resistance and soil resistivity generally occur in summer or autumn when there is high precipitation and temperature, while the maximum values occur in winter when the precipitation and temperature are low.

Therefore, the health status of the ground resistance was divided into five states—normal, attention, warning, abnormal, and critical—in order to quantitatively evaluate the health status of the butt resistance.

**Figure 9:**
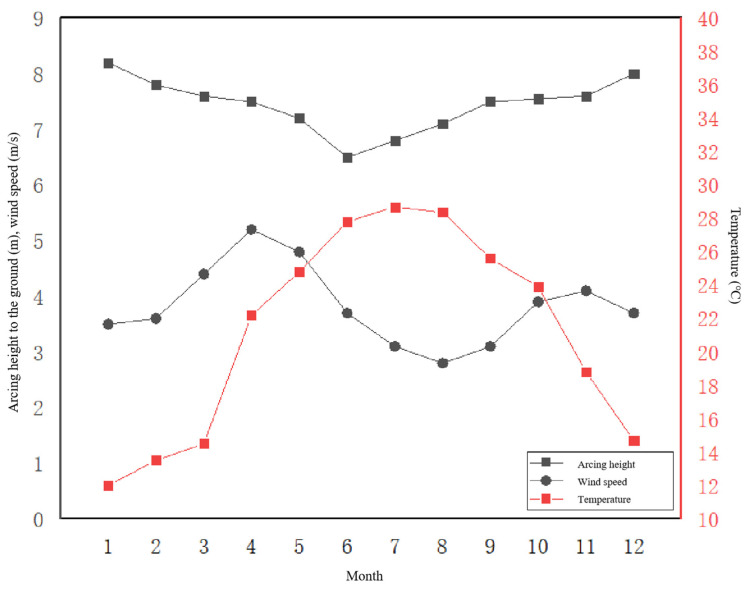
Monthly variation curves of arboreal height, air temperature, and wind speed to the ground.

**Figure 10:**
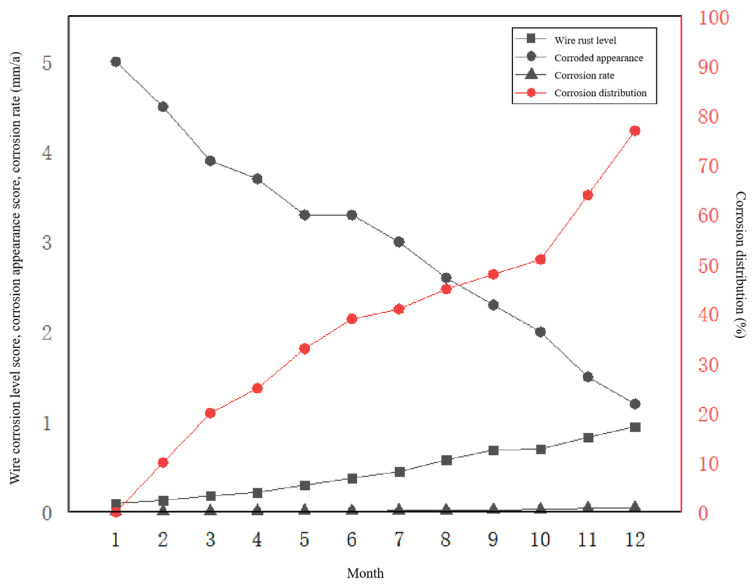
Annual variation curves of corrosion degree, corrosion rate, corrosion appearance, and corrosion distribution of the wire.

The health status of the arc of the grounding wire was taken as the main measurement standard, based on the characteristics of the variation of the arc. To ensure timeliness, the distance from the arc to the ground was used as the main evaluation index for the health status of the arc of the grounding wire, with air temperature and wind speed serving as auxiliary evaluation indices in Figure 9. The influence of air temperature, wind speed, and wind direction on the arc height was analysed using MATLAB simulation software. The results showed that as the air temperature and wind speed increase, the sag of the arc becomes larger and its distance to the ground decreases. According to the analysis results, combining these results with the characteristics of seasonal climate change, it was found that in the summer—when temperatures are higher and wind speeds are lower—the height of the arc from the ground reaches its trough in the monthly average trend for the year. In the winter, when temperatures are lower and wind speeds are higher, the height of the arc from the ground reaches its annual peak in January. In Figure 10 the trend of peaking and then valleying, and then valleying and peaking again, indicates that although temperature, wind speed, and sag are negatively correlated with ground height, the influence of temperature is significantly greater than that of wind speed.

Therefore, the arc health status of the wire was divided into five states—normal, attention, warning, abnormal, and critical—in order to achieve the quantitative evaluation of arc health status.

### 5.3. Determining the Dynamic Combination Weight of Transmission Lines’ Operating Condition Evaluation Index

According to the two updated weight results calculated in Section 5.1, the combined weight calculation method described in Section 4.1 was adopted to recalculate the dynamic combined weight of transmission lines’ operating status evaluation indicators, and the calculation results are shown in Table 12.

The operation status evaluation score of the 66 kV town line was calculated using the evaluation score calculation method described in Section 4.1 by combining the inspection record score of the 66 kV town line with the updated evaluation index weight based on the inspection defect parameters in the table. The calculation process is shown in Equation (23):

(23) 0.2×0.04+0.225×0.045+0.65×0.13+⋯…+0.03×0.006=3.892 

Similarly, the updated combined weights of the evaluation indicators were used to calculate the running status scores of other lines, and the scoring results are shown in Table 13.

As can be seen from the table, the line status score in 2019 was better than that in 2020 and 2021, indicating that the operation status of transmission lines has shown a downward trend with the increase in line operation time. In March 2021, the ranking of the evaluation status was 66 kV Chengbao line > 66 kV Town line > 66 kV Chengtai line > 66 kV Chenglan line, which is almost consistent with the sequence of line operation times, indicating that the later the transmission line is put into operation, the better its operational status. The score of the 66 kV platform line in the third evaluation showed an upward trend, indicating that the operation and maintenance effect of the line has a good effect and the state of the line was steadily improving. The third scores of the 66 kV Chengbao line and 66 kV Chenglan line showed a downward trend, indicating that the operation and maintenance of these lines should be properly strengthened to ensure the safety and stability of their state. The result of the onsite inspection is consistent with the calculation results of the evaluation model and has practical value.

## 6. Conclusions

In order to address the existing issues with the current methods for evaluating the operation status of ETLs, this paper improves the HSA–CRITIC process by building a complete measurement index system for the operation status of ETLs. The feasibility and innovation of the method were verified through theoretical analysis and calculation examples, comparing the results with industry standards and specifications. The ETL status was determined based on the HSA–CRITIC method in accordance with industry standards, and a comprehensive evaluation index system was established to complete the task. According to the problem existing in the index system, a method based on HSA–CRITIC was proposed. To overcome the issue that “the qualitative index cannot be calculated”, a general evaluation standard was formulated that unifies the qualitative and quantitative indices as quantitative indices for ease of calculation. Using the improved comprehensive evaluation method of the ETL condition, the operation status of six ETLs was evaluated using standard specifications, the HSA method, the improved HSA method, the CRITIC method, and the improved HSA–CRITIC method. The comparison of the evaluation results produced by these two methods shows that the improved HSA–CRITIC process combines the merits of the improved HSA method and the CRITIC method, while also avoiding the issue of weight imbalance and retaining experts’ subjective opinions. It is more in line with objective facts than traditional standard evaluation methods, and it has important guiding significance, especially for evaluating and providing early warning about the operation status of ETLs.

A large amount of objective data is required to calculate accurate weights, and the quality and quantity of objective data determine the accuracy of the evaluation method. In this project, data from six lines were calculated to verify the method’s feasibility, and high-quality data will be needed to improve the accuracy of the evaluation results. Although the improved HSA–CRITIC process can evaluate the operation states of each electric ETL, it lacks a unified evaluation standard for the status of a single line, which should be addressed in future research.

To address these issues, it will be necessary to combine more objective data to establish a database, develop a standard for the classification of ETLs’ operating status, and incorporate technologies such as big data and artificial intelligence in future research. This will allow for a more scientific and comprehensive assessment of the quality of the ETLs and provide a basis for early warning of their status, ensuring the safety and stability of the ETLs.

## Figures and Tables

**Table 1:** The meaning of the exponential scaling method.

Importance	Nine-Level Scale Value K	Exponential Scaling Value K-1 (a = 1.316)
Equally important	1	a^0^ = 1
More than equally important but less than slightly important	2	a^1^ = 1.316
Slightly important	3	a^2^ = 1.732
More than slightly important but less than Important	4	a^3^ = 2.279
Important	5	a^4^ = 3
More than obviously important but less than strongly important	6	a^5^ = 3.947
Strongly important	7	a^6^ = 5.194
More than strongly important but less than extremely important	8	a^7^ = 6.836
Extremely important	9	a^8^ = 9

**Table 2:** $R_{I}$ value table.

m	1	2	3	4	5	6	7	8	9	10	11	12
R_I_	0	0	0.52	0.89	1.12	1.26	1.36	1.41	1.46	1.49	1.52	1.54

**Table 3:** The scoring standard for line unit status [3].

The Scoring Standard for Line Unit Status [3]	Score
Normal status I	5
General state II	4
Attention status III	3
Abnormal state IV	2
Severe state V	1

**Table 4:** R_I_ value table.

Index	$T_{1}$	$T_{2}$	$T_{3}$	$T_{4}$	$T_{5}$	$T_{6}$	$T_{7}$	$T_{8}$
Weights	0.1078	0.3234	0.0948	0.1867	0.1419	0.0622	0.0473	0.0359

**Table 5:** Index weight of the appraise layer.

Index	$T_{51}$	$T_{52}$	$T_{53}$	$T_{54}$	$T_{55}$	$T_{56}$	$T_{57}$	$T_{58}$	$T_{59}$	$T_{510}$	$T_{511}$	$T_{512}$
Weights	0.1378	0.1378	0.1378	0.1378	0.1378	0.0918	0.047	0.047	0.047	0.047	0.0157	0.0157

**Table 6:** Overall weights of various indicators of the hardware appraise layer.

Index	$T_{51}$	$T_{52}$	$T_{53}$	$T_{54}$	$T_{55}$	$T_{56}$	$T_{57}$	$T_{58}$	$T_{59}$	$T_{510}$	$T_{511}$	$T_{512}$
Weights	0.0196	0.0196	0.0196	0.0196	0.0196	0.0130	0.0067	0.0067	0.0067	0.0067	0.0022	0.0022

**Table 7:** Criteria layer indicator weights.

Index	Foundation	Pole and Tower	Guide Ground	Insulator String	Gold Tools	Earthing Device	Ancillary Facilities	Channel Environment	Meteorological Environment
Weights	0.113	0.304	0.096	0.175	0.133	0.058	0.044	0.036	0.039

**Table 8:** Principles and characteristics of multi-index comprehensive early warning methods.

Early Warning Method	Principle	Characteristic
BP neural network	A self-learning network early warning method continuously updates the data optimisation model through self-learning until it reaches the optimal state [33]	These networks have good adaptability and can handle more complex problems, making them suitable for a wide range of applications [34]
Support-vector machine	According to statistical learning theory and the structural risk minimisation principles, limited samples strive to find the best balance between model complexity and learning ability in order to achieve the best generalisation ability [35]	They are particularly useful for solving small, nonlinear problems [36]
AHP-fuzzy comprehensive	For the index system, the AHP principle is used to determine the weight by comparing the importance of each index layer by layer, and the overall warning level is obtained by synthesising multiple index values using the membership theory in fuzzy mathematics [37]	Precise results for non-deterministic problems that are difficult to quantify [38]
Cloud model	The approach combines experts’ qualitative linguistic value descriptions with scientific quantitative calculation, allowing qualitative information expressed through linguistic values to be transformed into quantitative data or precise numerical values that can be effectively converted into appropriate qualitative linguistic values for analysis [39]	Taking into account randomness and ambiguity to effectively solve complex and fuzzy system problems [40]

**Table 9:** The distribution of score intervals.

Early Warning Level	Critical State	Abnormal State	Alert Status	Attention Status	Normal Status
Scoring interval	[0, c1]	[c1, c2]	[c2, c3]	[c3, c4]	[c4, 10]

**Table 10:** Numerical eigenvalues of the cloud model for ETL status warning level scoring intervals.

Early Warning Level	Scoring Interval	Cloud Model Digital Eigenvalues
Critical state	[0, 2]	(1, 0.33, 0.08)
Abnormal state	[2, 4]	(3, 0.33, 0.08)
Alert state	[4, 6]	(5, 0.33, 0.08)
Attention state	[6, 8]	(7, 0.33, 0.08)
Normal state	[8, 10]	(9, 0.33, 0.08)

**Table 11:** Comparison of ETL operation state appraisal results.

Line	Standard Specification		HSA		Improved HSA		CRITIC		Improved HSA–CRITIC	
	Evaluation Statue	Sort	Evaluation Score	Sort	Evaluation Score	Sort	Evaluation Score	Sort	Evaluation Score	Sort
#1	Notice	1	4.958	1	4.930	1	4.771	1	4.882	1
#2	Abnormal	5	4.861	4	4.834	4	4.120	4	4.620	3
#3	Notice	1	4.936	2	4.902	2	4.615	2	4.816	2
#4	Notice	1	4.669	6	4.688	6	4.233	3	4.551	5
#5	Notice	1	4.907	3	4.872	3	4.021	5	4.617	4
#6	Serious	6	4.753	5	4.731	5	3.798	6	4.451	6

**Table 12:** Updated weight table of evaluation index combination based on inspection defect parameters.

Criterion Layer	Evaluation Layer	Combined Weight Value
Foundation T_1_	Surface damage of tower foundation T_11_	0.040
	Foundation settlement T_12_	0.045
Pole and tower T_2_	Tilt of the tower T_21_	0.130
	Bending of the main wood T_22_	0.059
	Crack condition of tower rod T_23_	0.066
Guide ground T_3_	Corrosion, broken strands, damage, and flashover burns T_31_	0.035
	Foreign body hanging condition T_32_	0.037
	Abnormal vibration, dancing, and icing T_33_	0.012
	Arcing T_34_	0.008
Insulator string T_4_	Corrosion of iron cap and steel pin of insulator T_41_	0.030
	Insulator string tilt condition T_42_	0.029
	Breakage of insulators T_43_	0.050
	Zero value of porcelain insulator and self-detonation of glass insulator T_44_	0.038
	Lock pin defect T_45_	0.036
Gold tools T_5_	The defect of the instrument T_51_	0.051
	The condition of the fittings T_52_	0.039
	The displacement of the instrument T_53_	0.030
Earthing device T_6_	Grounding lead down connection T_61_	0.032
	Ground resistance value T_62_	0.040
	Grounding depth T_63_	0.027
Ancillary facilities T_7_	The defect of the lever plate T_71_	0.032
	Damage to bird control facilities T_72_	0.032
	Ladder and guardrail damage T_73_	0.007
Channel environment T_8_	Crossing distance T_81_	0.021
	Trees and buildings in the passageway T_82_	0.015
Meteorological environment T_9_	Temperature T_91_	0.031
	Humidity T_92_	0.013
	Wind speed T_93_	0.008
	Rainfall T_94_	0.006

**Table 13:** Order of transmission lines’ operating status in the Baicheng area.

Line Name	Inspection Time	Traditional Manual Scoring	Evaluation Score	Precedence Ranking
66 kV Chengbao line	2021.2	4	4.176	3
	2022.11	4	4.129	4
	2021.3	4	4.064	5
66 kV Chenglan line	2021.3	4	4.509	1
	2020.6	3	3.566	8
	2021.3	3	3.512	10
66 kV Chengtai line	2020.4	4	3.541	9
	2022.3	4	3.759	7
	2022.4	4	4.333	2
66 kV Town line	2022.3	4	3.892	6

## Data Availability

The data used to support the findings of this study are included in the article.
